# Cyclen-Based Cationic Lipids for Highly Efficient Gene Delivery towards Tumor Cells

**DOI:** 10.1371/journal.pone.0023134

**Published:** 2011-08-10

**Authors:** Qing-Dong Huang, Guo-Xing Zhong, Yang Zhang, Jiang Ren, Yun Fu, Ji Zhang, Wen Zhu, Xiao-Qi Yu

**Affiliations:** Key Laboratory of Green Chemistry and Technology (Ministry of Education), College of Chemistry, and State Key Laboratory of Biotherapy, West China Hospital, Sichuan University, Chengdu, People's Republic of China; University of South Florida College of Medicine, United States of America

## Abstract

**Background:**

Gene therapy has tremendous potential for both inherited and acquired diseases. However, delivery problems limited their clinical application, and new gene delivery vehicles with low cytotoxicity and high transfection efficiency are greatly required.

**Methods:**

In this report, we designed and synthesized three amphiphilic molecules (**L1**–**L3**) with the structures involving 1, 4, 7, 10-tetraazacyclododecane (cyclen), imidazolium and a hydrophobic dodecyl chain. Their interactions with plasmid DNA were studied via electrophoretic gel retardation assays, fluorescent quenching experiments, dynamic light scattering and transmission electron microscopy. The in vitro gene transfection assay and cytotoxicity assay were conducted in four cell lines.

**Results:**

Results indicated that **L1** and **L3**-formed liposomes could effectively bind to DNA to form well-shaped nanoparticles. Combining with neutral lipid DOPE, **L3** was found with high efficiency in gene transfer in three tumor cell lines including A549, HepG2 and H460. The optimized gene transfection efficacy of **L3** was nearly 5.5 times more efficient than that of the popular commercially available gene delivery agent Lipofectamine 2000™ in human lung carcinoma cells A549. In addition, since **L1** and **L3** had nearly no gene transfection performance in normal cells HEK293, these cationic lipids showed tumor cell-targeting property to a certain extent. No significant cytotoxicity was found for the lipoplexes formed by **L1**–**L3**, and their cytotoxicities were similar to or slightly lower than the lipoplexes prepared from Lipofectamine 2000™.

**Conclusion:**

Novel cyclen-based cationic lipids for effective in vitro gene transfection were founded, and these studies here may extend the application areas of macrocyclic polyamines, especially for cyclen.

## Introduction

Gene therapy, as a promising therapeutics to treat genetic or acquired diseases, has received significant attentions in the past several decades due to its advantages over traditional therapies [1]–[3]. The clinical success of gene therapy continues to remain critically dependent on the availability of safe and efficacious gene delivery reagents, popularly known as transfection vectors [4], [5]. Comparing to viral gene vectors, for the lesser immunogenic nature, robust manufacture ability to deliver large pieces of DNA, and ease of handling and preparation techniques, cationic lipids as non-viral vectors have attracted an upsurge of global interest in recent years for gene delivery [2], [6].

Since Felgner et al. first reported the utilization of unnatural diether-linked cationic lipid, N-[1-(2, 3-dioleyloxy) propyl]-N, N, N-trimethylammonium chloride (DOTMA), as a synthetic carrier to deliver gene into cells in 1987 [7], design and syntheses of more efficient cationic lipids [2], [6], [8] have been reported. The visible fruits of such intense global efforts toward developing safe and efficient cationic lipids for use in gene delivery are a number of commercially available cationic lipid-based transfection kits such as Lipofectamine and Lipofectamine 2000™ [9]. However, those cationic lipids were still far from the requirement of gene therapy because of their relative low transfection efficiency and potential cytotoxicity [10], [11]. Consequently, the development of novel nontoxic cationic structures high gene transfection efficiencies is of great importance.

Many cationic amphiphiles involving linear polyamines [12], [13] or branched polyamines [14] have been widely studied. Although cationic lipids using polyamine as the headgroups always show higher gene transfection efficiency than those containing one quaternary amine or single protonated amine [15]–[17], cationic lipids with long linear polyamine chains as headgroups might also have decreased gene delivery efficiency because of their relatively low binding ability towards DNA, which was resulted from self-folding of the linear lipopolyamine chains in the structure [18]. Since cyclic polyamines and branched polyamines are hard to self-fold, we postulated that using macrocyclic polyamines as the hydrophilic headgroups of cationic lipids may solve this problem. To the best of our knowledge, there is no example using cyclic polyamines as the hydrophilic headgroup of cationic lipid for gene delivery. Our previous studies revealed that some 1, 4, 7, 10-tetraazacyclododecane (cyclen) based cationic lipids [19] or linear [20] and reticular [21] polymers, which could transfer plasmid DNA into cells without use of extraneous agent. However, the transfection efficiency (TE) was not satisfying. It is necessary to further investigate the structure-activity relationship (SAR) of this type of cationic lipids. In addition, cationic lipids containing imidazolium polar heads [22], [23] have been reported to display higher transfection efficiency and reduced cytotoxicity when compared with classical quaternary ammonium cationic lipids.

**Figure 1:**
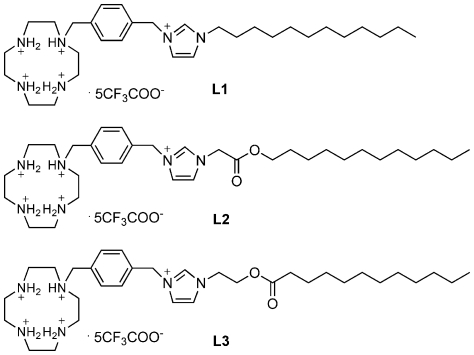
Molecular structures of target lipids (L1, L2 and L3).

In the present study, we designed and synthesized three cationic lipids **L1**–**L3** (Figure 1), which have the same hydrophilic headgroup (protonated cyclen and imidazolium salt). Their hydrophobic alkyl chains were connected to the headgroup via methylene (**L1**) or ester groups (**L2** and **L3**), and the sole structural difference between **L2** and **L3** is the orientation of the ester group. Their interactions with plasmid DNA and the properties of formed lipoplexes were examined. The in vitro transfection efficiencies towards four cell lines were investigated to study the SAR of this type of cationic lipids in gene delivery. Results indicated that tiny difference in the lipid structures might lead to essential distinction in the in vitro transfection efficiency, and **L3** has much higher luciferase reporter gene transfection efficiency than Lipofectamine 2000™ in human lung carcinoma cell line A549.

## Materials and Methods

### Materials

Unless stated otherwise, all chemicals and reagents were obtained commercially and used without further purification. Absolute chloroform (CHCl_3_) and dichloromethane (CH_2_Cl_2_) were distilled from calcium hydride (CaH_2_) and anhydrous acetonitrile (CH_3_CN) from phosphorus pentoxide. The precursor 1-bromomethyl-4-(4, 7, 10 tris(tertbutyloxycarbonyl)-1′, 4′, 7′, 10′-tetraazacyclododecan-1-yl-methylene)benzene (**3**) were prepared according to the literature [24]. IR spectra were recorded on a Shimadzu FTIR-4200 spectrometer as KBr pellets or thin films on KBr plates. The ^1^H NMR and ^13^C NMR spectra were measured on a Varian INOVA-400 spectrometer and the δ scale in parts per million was referenced to residual solvent peaks or internal tetramethylsilane (TMS). MS-ESI spectra data were recorded on a Finnigan LCQDECA and a Bruker Daltonics BioTOF mass spectrometer respectively. Fluorescence spectra were measured by a Horiba Jobin Yvon Fluoromax-4 spectrofluorometer. MTT (3-(4, 5-dimethylthiazol-2-yl)-2, 5-diphenyltetrazolium bromide) were purchased from Sigma-Aldrich (St. Louis, MO, USA). MicroBCA protein assay kit was obtained from Pierce (Rockford, IL, USA). Luciferase assay kit was purchased from Promega (Madison, WI, USA). Endotoxin free plasmid purification kit was purchased from TIANGEN (Beijing, China). The plasmids used in the study were pGL-3 (Promega, Madison, WI, USA) coding for luciferase DNA and pEGFP-N1 (Clontech, Palo Alto, CA, USA) coding for EGFP DNA. The Dulbecco's Modified Eagle's Medium (DMEM), RPMI 1640 Medium and fetal bovine serum were purchased from Invitrogen Corp. A549 (Human lung adenocarcinoma epithelial cells), HEK 293 (human embryonic kidney cells), Hep G2 (Human hepatocellular carcinoma cells), and H460 (Human lung cancer cells) cell lines were purchased from the American Type Culture Collection (ATCC).

### Synthesis of dodecyl chloroacetate (1b)

Chloroacetyl chloride (16.5 g, 150 mmol) in anhydrous dichloromethane (25 mL) was added dropwise to a stirred solution of 1-dodecanol (5 g, 30 mmol) and triethylamine (4.8 mL, 3.5 g, 34.5 mmol) in anhydrous dichloromethane (100 mL). The resulting reaction mixture was stirred for 4 h at room temperature (r. t.). Excess chloroacetyl chloride was removed by washing with a 5% solution of sodium hydrogen carbonate (50 mL×3) and brine (50 mL×1). The organic phase was dried over anhydrous magnesium sulfate, filtered, and rotary-evaporated. The resulting dodecyl chloroacetate (**1b**) was obtained as yellow oil, weighed 7.21 g, corresponding to 91% yield. IR (cm^−1^): 2926, 2855, 1760, 1463, 1415, 1381, 1307, 1177, 996, 790. ^1^H NMR (CDCl_3_, 400 MHz): δ 0.89 (t, *J* = 9.2 Hz, 3H, CH_2_ *CH_3_*), 1.27–1.37 (m, 16H, *(CH_2_)_8_*CH_3_), 1.67 (m, 2H, COCH_2_ *CH_2_*), 4.07 (s, 2H, Cl*CH_2_*CO), 4.19 (t, *J* = 8.8 Hz, 2H, CO*CH_2_*CH_2_). ^13^C NMR (CDCl_3_, 100 MHz): 14.10, 22.68, 25.75, 28.43, 29.17, 29.33, 29.46, 29.54, 29.61, 31.90, 40.93, 66.42, 167.39. ESI MS: 285(M+Na^+^).

### Synthesis of 2-chloroethyl laurate (1c)

The 2-chloroethyl laurate (**1c**) was synthesized according to the similar procedures described in reported literature [25]. Briefly, to pure thionyl chloride (25 mL, 42 g, 352 mmol) was added lauric acid (10 g, 50 mmol) gradually. The resulting mixture was heated at reflux for about 1 h. The excess thionyl chloride was removed under reduced pressure. Lauroyl chloride was obtained as a brown oil liquid (9.2 g, 40 mmol) which was directly used in the next step without further purification. The obtained lauroyl chloride (9.2 g, 40 mmol) in anhydrous dichloromethane (15 mL) was added dropwise to a stirred solution of 2-chloroethyl alcohol (2.7 g, 30 mmol) and triethylamine(4.8 mL, 3.5 g, 35 mmol) in anhydrous dichloromethane (50 mL). The resulting reaction mixture was stirred for 4 h at room temperature and then washed with 5% solution of sodium hydrogen carbonate (30 mL×3) and brine (30 mL×1). The organic phase was dried over anhydrous magnesium sulfate, filtered, and rotary-evaporated to get crude 2-chloroethyl laurate as brown oil. Pure 2-chloroethyl laurate was obtained as a yellow liquid by flash chromatography over silica (petroleum ether/ethyl acetate, 20/1), weighed 6.4 g, corresponding to 81% yield. IR (cm^−1^): 3467, 2926, 2679, 1742, 1461, 1384. ^1^H NMR (CDCl_3_, 400 MHz): δ 0.88 (t, *J* = 13.6 Hz, 3H, CH_2_ *CH_3_*), 1.22–1.30 (br, 16H, *(CH_2_)_8_*CH_3_), 1.64 (m, 2H, COCH_2_ *CH_2_*), 2.35 (t, *J* = 15.2 Hz, 2H, CO*CH_2_*), 3.68 (t, *J* = 11.6 Hz, 2H, *CH_2_*OCO), 4.33 (t, *J* = 11.6 Hz, 2H, Cl*CH_2_*). ^13^C NMR (CDCl_3_, 100 MHz): 14.08, 22.66, 24.87, 29.07, 29.22, 29.31, 29.43, 29.57, 31.89, 34.06, 41.61, 63.77, 173.45. ESI MS: 263 (M+H^+^), 285 (M+Na^+^).

### General Synthesis of imidazole derivatives (2a, 2b and 2c)

Halogen derivatives(**1a**, **1b** or **1c**) (22.6 mmol) was added to a solution of imidazole (18.46 g, 271 mmol), sodium carbonate (2.4 g, 23 mmol), potassium iodine (0.2 g, 1.2 mmol) in anhydrous chloroform (30 mL) and dimethyl sufloxide (20 mL). The reaction mixture was heated at 90°C for 10 h, then chloroform (150 mL) was added. The resulting reaction mixture was washed with 5% solution of sodium hydrogen carbonate (100 mL×3) and brine (100 mL×1). The organic phase was dried over anhydrous magnesium sulfate, filtered, and rotary-evaporated to get crude residue which were purified by flash, chromatography over silica (ethyl acetate) to obtain the corresponding imidazole derivatives **2a**, **2b** and **2c**.

N-Dodecylimidazole (**2a**): yellow oil liquid, yield 88%. IR (cm^−1^): 3388, 3107, 2925, 2854, 1506, 1465, 1376. ^1^H NMR (CDCl_3_, 400 MHz): δ 0.88 (t, *J* = 13.6 Hz, 3H, CH_2_ *CH_3_*), 1.25–1.30 (m, 18H, *(CH_2_)_9_*CH_3_), 1.77 (m, 2H, COOCH_2_ *CH_2_*), 3.92 (t, *J* = 14 Hz, 2H, COO*CH_2_*CH_2_), 6.90 (d, 1H, imidazole-H), 7.05 (d, 1H, imidazole-H), 7.48 (s, 1H, imidazole-H). ^13^C NMR (CDCl_3_, 100 MHz): 14.13, 22.69, 26.55, 29.07, 29.33, 29.43, 29.52, 29.60, 31.09, 31.90, 47.04, 118.76, 129.34, 137.06. ESI MS: 237 (M+H^+^), 259 (M+Na^+^).

1H-Imidazole-1-acetic acid, dodecyl ester (**2b**): white solid, yield 90%. IR (cm^−1^): 3472, 3117, 2958, 2921, 2851, 1744, 1512, 1210. ^1^H NMR (CDCl_3_, 400 MHz): δ 0.88 (t, *J* = 13.6 Hz, 3H, CH_2_ *CH_3_*), 1.26–1.30 (br, 18H, *(CH_2_)_9_*CH_3_), 1.64 (m, 2H, COOCH_2_ *CH_2_*), 4.17 (t, *J* = 13.6 Hz, 2H, COO*CH_2_*CH_2_), 4.70 (s, 2H, N*CH_2_*COO), 6.96 (d, 1H, imidazole-H), 7.10 (d, 1H, imidazole-H), 7.51 (s, 1H, imidazole-H). ^13^C NMR (CDCl_3_, 100 MHz): 14.10, 18.43, 22.67, 25.72, 28.39, 29.13, 29.32, 29.44, 29.52, 29.60, 31.89, 48.06, 58.13, 66.23, 119.94, 129.65, 137.91, 167.47. ESI MS: 295.4 (M+H^+^).

Dodecanoic acid, 2-(1H-imidazol-1-yl)ethyl ester (**2c**): yellow oil liquid, yield 86%. IR (cm^−1^): 3381, 3112, 2925, 2854, 1740, 1507, 1461. ^1^H NMR (CDCl_3_, 400 MHz): δ 0.88 (t, *J* = 13.6 Hz, 3H, CH_2_ *CH_3_*), 1.22–1.40 (br, 16H, *(CH_2_)_8_*CH_3_), 1.60 (m, 2H, COOCH_2_ *CH_2_*), 2.31 (t, *J* = 15.2 Hz, 2H, COO*CH_2_*CH_2_), 4.20 (t, *J* = 10.4 Hz, 2H, N*CH_2_*CH_2_O), 4.33 (t, *J* = 10.4 Hz, 2H, *CH_2_*OCO), 6.99 (d, 1H, imidazole-H), 7.10 (d, 1H, imidazole-H), 7.54 (s, 1H, imidazole-H). ^13^C NMR (CDCl_3_, 100 MHz): 14.09, 22.65, 24.76, 29.04, 29.18, 29.30, 29.40, 29.55, 31.87, 34.01, 45.84, 62.97, 173.31. ESI MS: 295.2 (M+H^+^).

### General synthesis of precursors (4a, 4b and 4c)

A solution of imidazole derivatives (**2a**, **2b** or **2c**) (5 mmol) and 1-bromomethyl-4-(4, 7, 10-tris-(tert-butyloxycarbonyl)-1, 4, 7, 10-tetraazacyclododecan-1-yl-methyl)benzene (**3**, 0.66 g, 10 mmol) in anhydrous acetonitrile (20 mL) was refluxed at 90°C over a period of 48–96 h, after which TLC indicated the disappearance of the starting material imidazole derivatives. Then reaction mixture was cooled and the acetonitrile was rotary-evaporated to yield a yellow residue, which was purified by chromatography over silica (dichloromethane/methanol, 20∶1) to obtain white foamy solid in 50–68% yield.

Compound **4a**: IR (cm^−1^): 2926, 2854, 1692, 1461, 1415, 1366, 1249, 1160. ^1^H NMR (CDCl_3_, 400 MHz): δ 0.87 (t, *J* = 6.8 Hz, 3H, CH_2_ *CH_3_*), 1.25–1.47 (br, 45H, (N-*Boc*)_3_ and *(CH_2_)_9_*CH_3_), 1.92 (m, 2H, NCH_2_ *CH_2_*), 2.64 (m, 4H, cyclen-*H*), 3.30–3.57 (br, 12H, cyclen-*H*), 3.72 (s, 2H, Ar*CH_2_*-cyclen), 4.30 (t, *J* = 15.2 Hz, 2H, imidazole-*CH_2_*CH_2_), 5.59 (s, 2H, Ar*CH_2_*-imidazole), 7.12 (d, 1H, imidazole-*H*), 7.17 (d, 1H, imidazole-*H*), 7.30–7.32 (d, 2H, Ar-*H*), 7.40–7.42 (d, 2H, Ar-*H*). HRMS (C_46_H_79_N_6_O_6_): Calcd. 811.6061; Found. 811.6046.

Compound **4b**: IR (cm^−1^): 3365, 2926, 2855, 1750, 1691, 1563, 1461, 1415, 1365, 1249, 1165. ^1^H NMR (CDCl_3_, 400 MHz): δ 0.88 (t, *J* = 9.2 Hz, 3H, CH_2_ *CH_3_*), 1.26–1.56 (br, 45H, *(CH_2_)_9_*CH_3_ and (N-*Boc*)_3_), 1.66 (m, 2H, COCH_2_ *CH_2_*), 2.48–2.64 (m, 4H, PhN*(CH_2_*)_2_), 3.31–3.80 (m, 14H, cyclen-*H* and Ar-*CH_2_*-cyclen), 4.19 (t, *J* = 7.2, 2H, COO*CH_2_*), 5.44 (s, 2H, Ar-*CH_2_*-imidazole), 5.52 (s, 2H, imidazole-*CH_2_*-COO), 7.31–7.47 (m, 4H, Ar-*H*), 7.54 (s, 1H, imidazole-*H*), 7.56 (s, 1H, imidazole-*H*), 10.39 (s, 1H, imidazole-*H*). ^13^C NMR (CDCl_3_, 100 MHz): 14.10, 22.65, 25.69, 25.75, 28.33, 28.45, 28.65, 29.16, 29.23, 29.43, 29.45, 29.56, 29.58, 29.60, 31.88, 32.79, 50.28, 53.33, 62.93, 67.10, 79.60, 123.74, 128.90, 166.07. ESI MS: 869 (M^+^), 812 (M^+^-CH_2_CH_2_ CH_2_CH_3_).

Compound **4c**: IR (cm^−1^): 3431, 2973, 2928, 2855, 1741, 1690, 1563, 1461, 1415, 1366, 1250, 1159. ^1^H NMR (CDCl_3_, 400 MHz): δ 0.87 (t, *J* = 13.6 Hz, 3H, CH_2_ *CH_3_*), 1.11–1.33 (br, 16H, *(CH_2_)_8_*CH_3_), 1.43–1.57 (br, 27H, (N-*Boc*)_3_), 1.64 (m, 2H, COOCH_2_ *CH_2_*), 2.33 (t, *J* = 15.2 Hz, 2H, COO*CH_2_*CH_2_), 2.64 (m, 4H, cyclen-*H*), 3.30–3.69 (br, 12H, cyclen-*H*), 3.76 (s, 2H, Ar*CH_2_*-cyclen), 4.51 (t, *J* = 9.6 Hz, 2H, *CH_2_*OCO), 4.76 (t, *J* = 8.8 Hz, 2H, N*CH_2_*CH_2_OCO), 7.31 (d, 2H, Ar-*H*), 7.41 (d, 1H, imidazole-*H*), 7.48 (d, 2H, Ar-*H*), 7.70 (d, 1H, imidazole-*H*), 10.44 (d, 1H, imidazole-*H*). ^13^C NMR (CDCl_3_, 100 MHz): 22.04, 22.49, 24.58, 28.35, 28.54, 28.94, 29.15, 29,32, 29.45, 30.75, 31.72, 33.80, 47.56, 49.01, 49.73, 50.26, 52.76, 52.96, 54.24, 54.95, 55.95, 60.01, 60.96, 61. 98, 79.29, 79.36, 121.87, 123.04, 128.83, 130.95, 131.80, 137.14, 138.26, 155.29, 155.63, 172.87, 206.63. ESI MS: 869.9 (M^+^).

### General Synthesis of L1, L2 and L3

Precursor (**4a**, **4b** or **4c**, 1 mmol) was suspended in anhydrous dichloromethane (10 mL), and then a solution of trifluoroacetic acid (5 mL) in anhydrous dichloromethane (5 mL) was added dropwise under ice-cold water and N_2_ atmosphere. The obtained mixture was stirred at room temperature for 6 h. The solvent and excess trifluoroacetic acid were removed under reduced pressure to obtain the final lipids as a yellow liquid in 98–100% yield.

Lipid **L1**: IR (KBr, cm^−1^): 3417, 2925, 1794, 1687, 1562, 1515, 1460, 1354, 1269, 1199, 1044, 934, 823, 747. ^1^H NMR (CDCl_3_, 400 MHz): δ 0.87 (t, *J* = 9.9 Hz, 3H, CH_2_ *CH_3_*), 1.26 (br s, 18H, *(CH_2_)_9_*CH_3_), 1.83 (m, 2H, NCH_2_ *CH_2_*), 2.89–2.91 (m, 4H, PhN*(CH_2_*)_2_), 3.04 (m, 4H, PhN(CH_2_ *CH_2_*)_2_), 3.19–3.25 (m, 8H, N(*CH_2_CH_2_*)_2_), 3.86 (s, 2H, Ar*CH_2_*N(CH_2_CH_2_)_2_), 4.20 (t, *J* = 10.8 Hz, 2H, (CH)(CH)N*CH_2_*CH_2_), 5.41 (s, 2H, Ar*CH_2_*N(CH)(CH)), 7.43 (m, 4H, CH_2_ *Ar*CH_2_), 7.52 (d, 1H, imidazole-H), 7.54 (d, 1H, imidazole-H), 9.01 (s, 1H, imidazole-H). ^13^C NMR (DMSO-d_6_): 14.40, 22.55, 26.04, 28.81, 29.16, 29.28, 29.38, 29.46, 29.98, 31.14, 31.75, 42.59, 42.71, 45.22, 47.74, 48.80, 49.47, 121.10, 122.23, 123.05, 123.31, 127.91, 128.01, 128.85, 129.77, 130.10, 131.03, 134.37, 134.45, 134.48, 134.54, 135.85, 136.66, 206.89. HRMS (C_31_H_55_N_6_^+^): Calcd. 511.4488; Found 511.4442.

Lipid **L2**: IR (KBr, cm^−1^): 3411, 2926, 2855, 1749, 1684, 1565, 1460, 1201, 1170, 1128, 828, 799, 719. ^1^H NMR (DMSO-d_6_, 400 MHz): δ 0.86 (t, *J* = 12.8 Hz, 3H, CH_2_ *CH_3_*), 1.16–1.25 (br s, 18H, (CH_2_)_9_), 1.60 (m, 2H, COOCH_2_ *CH_2_*), 2.73 (m, 4H, PhN*(CH_2_*)_2_), 2.85–2.92 (m, 4H, PhN(CH_2_ *CH_2_*)_2_), 3.06–3.14 (m, 8H, N(*CH_2_CH_2_*)_2_), 3.76 (s, 2H, Ar*CH_2_*N(CH_2_CH_2_)_2_), 4.14 (t, 2H, COO*CH_2_*CH_2_), 5.30 (s, 2H, Ar*CH_2_*N(CH)(CH)), 5.53 (s, 2H, (CH)(CH)N*CH_2_*CO), 7.34–7.51 (m, 4H, CH_2_ *Ar*CH_2_), 7.80 (s, 1H, imidazole-H), 7.88 (s, 1H, imidazole-H), 9.37 (s, 1H, imidazole-H). ^13^C NMR (DMSO-d_6_): 22.51, 25.66, 28.40, 29.05, 29.13, 29.35, 29.41, 29.43, 29.47, 31.72, 39.42, 39.62, 39.83, 40.04, 42.57, 42.75, 45.22, 47.77, 50.14, 52.24, 55.66, 66.23, 122.73, 124.71, 128.84, 131.03, 134.21, 136.62, 137.95, 167.30. HRMS (C_33_H_57_N_6_O_2_^+^): Calcd. 569.4543; Found 569.4468.

Lipid **L3**: IR (KBr, cm^−1^): 2926, 2855, 1714, 1673, 1460, 1410, 1199, 1137, 832, 800, 721. ^1^H NMR (DMSO-d_6_, 400 MHz): δ 0.78 (t, *J* = 13.2 Hz, 3H, CH_2_ *CH_3_*), 1.09–1.22 (br, 16H, *(CH_2_)_8_*CH_3_), 1.38 (m, 2H, COOCH_2_ *CH_2_*), 2.21 (t, *J* = 14.8 Hz, 2H, OCO*CH_2_*CH_2_), 2.65 (m, 4H, PhN*(CH_2_*)_2_), 2.78–2. 82 (m, 4H, N(CH_2_ *CH_2_)_2_*), 2.99–3.18 (m, 8H, N(CH_2_CH_2_)_2_), 3.85 (s, 2H, Ar*CH_2_*N(CH_2_CH_2_)_2_), 4.33 (t, *J* = 9.6 Hz, 2H, *CH_2_*OCO), 4.43 (t, *J* = 8.8 Hz, 2H, N*CH_2_*CH_2_O), 5.40 (s, 2H, Ar*CH_2_*N(CH)(CH)), 7.30–7.36 (m, 4H, CH_2_ *Ar*CH_2_), 7.77 (s, 1H, imidazole-H), 9.36 (s, 1H, imidazole-H). ^13^C NMR (DMSO-d_6_): 22.54, 24.66, 28.82, 29.10, 29.15, 29.31, 29.44, 31.74, 33.62, 39.36, 39.57, 39.78, 39.99, 42.52, 42.63, 45.14, 47.58, 48.71, 52.10, 55.43, 59.81, 62.22, 123.01, 123.72, 128.76, 131.01, 134.35, 136.35, 137.23, 172.98. HRMS (C_33_H_57_N_6_O_2_^+^): Calcd. 569.4543; Found 569.4540.

### Amplification and purification of plasmid DNA

pGL-3 and pEGFP-N1 plasmids were used. The former one was used as the luciferase reporter gene which was transformed in JM109 Escherichia coli, and the latter one was used as the enhanced green fluorescent protein reporter gene which was transformed in E. coli DH5α. Both plasmids were amplified in E. coli grown in LB media at 37°C and 220 rpm overnight. The plasmids were purified by an EndoFree TiangenTM Plasmid Kit. Then the purified plasmids were dissolved in TE buffer solution and stored at −20°C. The integrity of plasmids was confirmed by agarose gel electrophoresis. The purity and concentration of plasmids were determined by the ratio of ultraviolet (UV) absorbances at 260 nm to 280 nm.

### Preparation of cationic liposome

All cationic lipids were stored as stock solutions in anhydrous chloroform at −20°C under argon. Individual cationic lipid (0.005 mmol) or its mixture with DOPE in the desired mole ratio was dissolved in anhydrous chloroform (5 mL) in autoclaved glass vials. Thin films were made by slowly rotary-evaporating the solvent at room temperature. Last traces of organic solvent were removed by keeping these films under vacuum above 8 h. The dried films and freshly autoclaved tris buffer (10 mM, PH 7.4) was preheated to 70°C, then the tris buffer (5 mL) was added to the films such that the final concentration of the cationic lipid was 1 mM. The mixtures were vortexed vigorously until the films were completely resuspended. Sonication of these suspensions for 20 min in a bath sonicator at 60°C afforded the corresponding cationic liposomes which were stored at 4°C.

### Preparation of liposome/pDNA complexes (lipoplexes)

To prepare the cationic lipid/pDNA complexes (lipoplexes), various amounts of cationic lipid in liposome solutions were mixed with a constant amount of DNA by pipetting thoroughly at various N/P ratios, and incubated for 30 min at room temperature. The theoretical N/P ratio of lipid to DNA represents the ratio of charge on cationic lipid (in mol) to nucleotide base molarity and was calculated by considering the average nucleotide mass of 350.

### Gel retardation assay

To determine the formation of liposome/DNA complex (lipoplexes), lipoplexes of various N/P ratios ranging from 0 to 8 were prepared as described above. Constant amount of 5 µg DNA was used here. 10 µL of each lipoplexes solution were electrophoresed on the 1% (W V-1) agarose gel containing Gold view and Tris-acetate (TAE) running buffer at 110 V for 30 min. DNA was visualized with a UV lamp using a BioRad Universal Hood II.

### Ethidium bromide intercalation assay

Fluorescence emission due to ethidium bromide (EB) at 605 nm was monitored in a Fluoromax-4 spectrofluorimeter (Excitation wavelength was 497 nm with 3 nm slit widths for both excitation and emission). Typically fluorescence emission was measured for EB (5 µM) in 10 mM Tris, pH 7.4 buffer. To this solution CT-DNA (11 µM) was added and the fluorescence emission due to EB upon intercalative complexation with DNA was measured again at 25°C. Then aliquots of a given cationic liposome (0.68 µM) were added into the EB/CT-DNA solution for further measurement. If F_0_ is the fluorescence intensity (FI) of unintercalated and F_max_ is the FI of fully intercalated EB, and F_x_ is the FI for a given concentration of liposome, then %FI was calculated as %FI = (F_x_−F_0_)/(F_max_−F_0_)×100.

### Dynamic light scattering (DLS)

Particle size and zeta-potential of liposomes or lipoplexes at various N/P ratio were measured by a dynamic light scattering system (Zetasizer Nano ZS, Malvern Instruments Led) at 25°C.

### Transmission electron microscopy

The morphology and particle size distribution of the liposomes and lipoplexes were examined using a TecnaiG^2^ F20 Transmission Electron Microscope (TEM) operated at 200 keV. A drop of lipoplex suspensions at optimized lipid/DOPE/DNA ratios (as in the final luciferase expression assay) was added to carbon coated 400-mesh copper grids, and allowed to adsorb for about 30 s followed by negative staining with a drop of 0.5% v/v phosphotungstic acid. Excess stain was removed after 1 min and the grids were air dried at room temperature before observing under TEM.

### Cell culture

A549 (Human lung adenocarcinoma epithelial cells), Hep G2 (Human hepatocellular carcinoma cells), and H460 (Human lung cancer cells) cell lines were incubated in RPMI medium 1640 containing 10% fetal bovine serum (FBS) and 1% antibiotics(penicillin-streptomycin, 10000 U mL-1), HEK 293 (human embryonic kidney cells) were incubated in Dulbecco's Modified Eagle's Medium (DMEM) containing 10% fetal bovine serum (FBS) and 1% antibiotics. All cells were maintained at 37°C under an atmosphere of 5% CO2–95% air.

### Transfection procedure

24-well plates were seeded with 45000–60000 cell/well in 500 µL antibiotic-free media 24 h before transfection in order to obtain about 80%–90% confluent cultures at the time of transfection. For the preparation of lipoplexes applied to cells, various amounts of liposomes and DNA were serially diluted separately in both serum and antibiotic-free RPMI1640 or DMEM culture medium, then the DNA solutions were added into liposome solutions and mixed briefly by pipetting up and down several times, after which the mixtures were incubated at room temperature for about 30 min to obtain lipoplexes of desired N/P ratios, the final lipoplexes volume was 200 µL and the DNA was used at a concentration of 2 µg/well unless otherwise noted. After 30 min of complexation, old cell culture medium was removed from the wells, cells were washed once with serum-free RPMI1640 or DMEM, and the above 200 µL lipoplexes were added to each well. The plates were then incubated for 4 h at 37°C in a humidified atmosphere containing 5% CO_2_. At the end of incubation period, medium was removed and 500 µL of fresh RPMI1640 or DMEM medium containing 10% FBS was added to each well. Plates were further incubated for a period of 24 h before checking the reporter gene expression.

For fluorescent microscopy assays, cells were transfected by complexes containing pEGFP-N1. After 24 h incubation, the microscopy images were obtained at the magnification of 40× and recorded using Viewfinder Lite (1.0) software.

For luciferase assays, cells were transfected by complexes containing pGL-3. For a typical assay in a 24-well plate, 24 h post-transfection as described above, the old medium was removed from the wells and the cells were washed twice with 500 µL of pre-chilled PBS. According to Luciferase assay kit (promega) manufacture, 100 µL of 1× cell lysis buffer diluted with PBS was then added to each well, and the cells were lysed for 30 min in a horizontal rocker at room temperature. The cell lysate was transferred completely to Eppendorf tubes and centrifuged (4000 rpm, RT) for 2 min; the supernatant was transferred to Eppendorf tubes and stored in ice. For the assay, 20 µL of this supernatant and 100 µL of luciferase assay substrate (Promega) were used. The lysate and the substrate were both thawed to RT before performing the assay. 20 µL lysate was added to 100 µL substrate and the luciferase activity was measured in a luminometer (Thermo Scientific Fluoroskan Ascent FL, Thermo, U.S.A.) in standard kinetic-luminescence mode. The integration time of measurement was 10000 ms. A delay of 2 s was given before each measurement. Each well was measured for 8 times. The protein concentration in the cell lysate supernatant was estimated in each case with Thermo Modified Lowry Protein Assay Kit (Thermo, Rockford, IL, USA). Comparison of the transfection efficiencies of the individual lipids was made based on data for luciferase expressed as relative light units (RLU)/mg of protein. All the experiments were done in triplicates, and results presented are the average of at least two such independent experiments done on the same days.

### Cytotoxicity assays

Toxicity of lipoplexes toward A549, Hep G2, H460 and HEK293 cells was determined using MTT (3-(4, 5-dimethylthiazol-2-yl)-2, 5-diphenyltetrazolium bromide) reduction assay following literature procedures [26], [27]. About 4500–6000 cells/well were seeded into 96-well plates. After 24 h, optimized lipid/DOPE formulations were complexed with 0.5 µg of DNA at various N/P ratios for 30 min. 100 µL of lipoplexes were added to the cells in the absence of serum. After 4 h of incubation, lipoplex solutions were removed and 200 µL of media with 10% FBS was added. After 24 h, 20 µL of MTT solution was added and the cells were incubated further for 4 h. Blue formazan crystals were seen at well when checked under microscope. Media were removed and 150 µL of DMSO was added per well and then plates was incubated on a shaker for 10 min at room temperature to dissolve blue crystal. The absorbance was measured using a microtiter plate reader. The % viability was then calculated as [A_490_(treated cells)-background]/[A_490_(Untreated cells)-background] ×100. The cytotoxicity of lipoplexes prepared from Lipofectamine 2000™ was used as control, which was prepared based on the standard conditions specified by the manufacturer.

## Results and Discussion

### Chemistry

Three novel cyclen-based cationic lipids (**L1**–**L3**) were designed and synthesized by using protonated cyclen and imidazolium salt group as the headgroup of cationic lipids (As shown in Figure 1). All cationic lipids have the same hydrophilic headgroup (protonated cyclen and imidazolium salt). Their hydrophobic alkyl chains were connected to the headgroup via methylene (**L1**) or ester groups (**L2** and **L3**), and the sole structural difference between **L2** and **L3** is the orientation of the ester group. Cyclen derivatives, which was extensively studied in our group for their synthesis and interaction with DNA [21], [28]–[31] was introduced here because protonated cyclen has pH-dependent charged nitrogen, centralized amine density and non-folded conformations which would make against their interactions with DNA [18]. All cationic lipids were synthesized according to Scheme S1. Halogenated derivatives (**1b** and **1c**) were firstly prepared through ester reaction between corresponding alcohols (1-dodecanol or 2-chloroethyl alcohol) and acyl chloride (chloroacetyl chloride or lauroyl chloride). Lauroyl chloride was prepared as described in literature [25]. With potassium iodide as catalyst, halogen derivatives **1** reacted with imidazole in the presence of sodium carbonate under 90°C in chloroform and dimethyl sulfoxide (DMSO) for about 10 h to give imidazole derivatives **2**. Subsequently reaction between **2** and compound **3** in acetonitrile led to the Boc-protected cyclen-imidazolium compounds **4**. The final products **L1–L3** were obtained by removing the Boc groups in methylene dichloride containing trifluoroacetic acid. All lipid compounds were characterized by ^1^H-NMR, ^1^C-NMR, ESI-MS and IR.

### Physicochemical characterizations of liposome/DNA complexes (lipoplexes)

**Figure 2:**
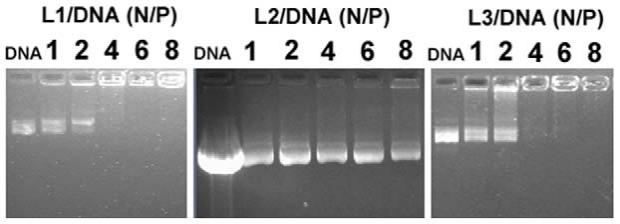
Electrophoretic gel retardation assays of liposomes/DNA complexes (lipoplexes). The N/P ratios are indicated at the top of each lane. All lipid/DOPE ratios were 1 ∶ 2 and the concentration of DNA was 5 µg/well.

The formation of lipoplexes between cationic liposomes and DNA was firstly confirmed by gel retardation assay. The electrophoretic gel patterns revealed an interesting feature. As shown in Figure 2, **L1** and **L3**-based liposome were capable of completely inhibiting electrophoretic mobility of DNA at the N/P ratios of 4. In contrast, **L2** was found to be unable to inhibit the electrophoretic mobility of DNA even at high N/P ratios of 8, indicating a relatively weaker affinity of **L2** to DNA.

**Figure 3:**
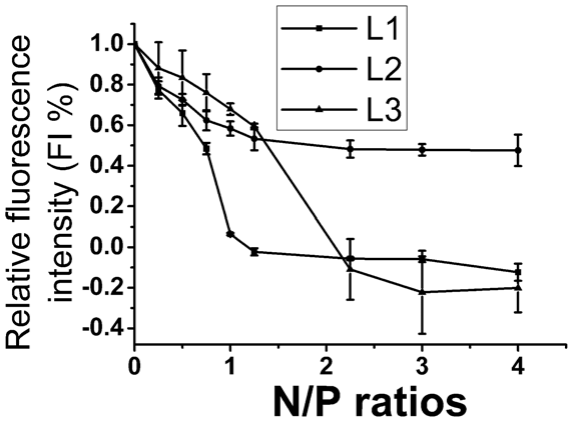
Release of the ethidium bromide (EB) from the CT (calf thymus) DNA-EB complexes upon addition of the liposomes (L1–L3) at different N/P ratios. All lipid/DOPE ratios were 1 ∶ 2 and the concentration of CT-DNA was 11 µM.

Ethidium bromide (EB) intercalation assay was also conducted to further investigate the interaction between liposomes and DNA. As shown in Figure 3, **L2**-based liposome showed a relatively weak and linear decrease of EB fluorescence intensity as a function of N/P ratios, which was according with the gel electrophoresis results that indicated its relatively weaker interaction with DNA. However, **L1** and **L3**-based liposome showed a sharp decrease of EB fluorescence at N/P ratio of 1 and 2, respectively. Thus, in accordance with the gel electrophoresis data, it was confirmed that **L1** and **L3** could bind strongly to DNA by showing a significant level of DNA condensation. The order of binding abilities of those liposomes to DNA is **L1**>**L3**>**L2**. By taking into account that three cationic lipids have the same hydrophilic head groups hydrophobic alkyl chains, the order of binding abilities is probably connected with the order of hydrophobicity of linker group (**L1**>**L3**>**L2**), which in turn favors their self-assembly and their cooperative interaction with DNA [32].

**Figure 4:**
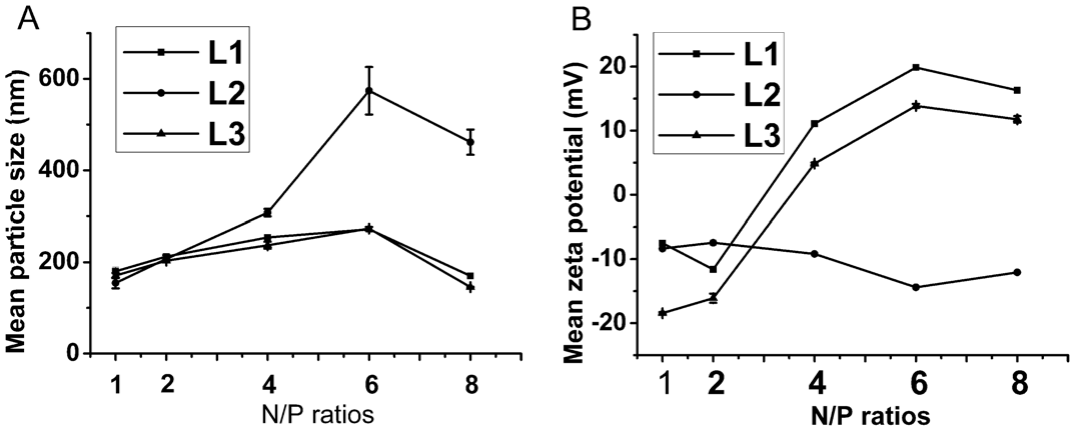
Particle sizes (A) and zeta-potentials (B) of lipid/DOPE/DNA complexes at different N/P ratios by DLS. All lipid/DOPE ratios were 1 ∶ 2 and the concentration of DNA was 5 µg/well.

According to DLS data shown in Figure 4, **L1** and **L3**-based liposomes could self-assemble into nanoparticles (around 100–300 nm) with DNA at various N/P ratios (as in gel retardation assay). In comparison, the particles of **L2**/DNA had bigger size around 400–550 nm especially at relatively higher N/P ratio of 6 and 8 (Figure 4A), which is probably due to the weak interaction between **L2** and DNA. The negative zeta potentials of **L2**/DNA at various N/P ratios (from 1–8) also demonstrated their invalid self-assembly (Figure 4B). In contrast, the zeta potentials of **L1** and **L3**/DNA lipoplexes reversed to positive (from +5 mV to +20 mV) at N/P ratios from 4 to 8 (Figure 4B). The zeta potential results were conformed to the above gel retardation assay and EB intercalation assay.

**Figure 5:**
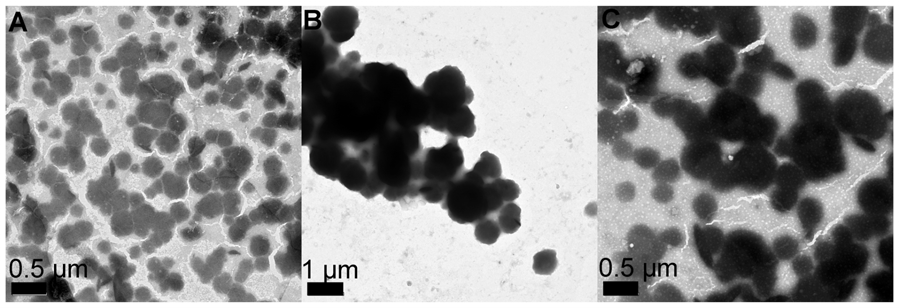
Representive transmission electron micrographs of the lipoplexes prepared from L1 (A), L2 (B) and L3 (C). Lipid/DOPE ratios were 1 ∶ 2 and concentration of DNA was 2 µg, the N/P ratio was 6 for all lipoplexes.

To get further insights into the formed lipoplexes, we also performed electron microscopy of lipoplexes at lipid/DOPE/ratio of 1 ∶ 2 with N/P ratio of 6. Representive electron micrographs of lipoplexes were shown in Figure 5. The lipoplexes formed from **L1** and **L3** showed homogeneous spherical particles with the sizes in the range of 200 to 400 nm. Meanwhile, the lipoplex prepared from **L2** formed bigger particles (around 800 nm) and showed a tendency to aggregate. These results also suggested that comparing to **L1** and **L3**, lipid **L2** is not a good candidate for DNA condensation. It was also demonstrated that the little difference in structure (ester orientation) might have large influence on the liposome formation and subsequent DNA affinity.

### In vitro transfection

**Figure 6:**
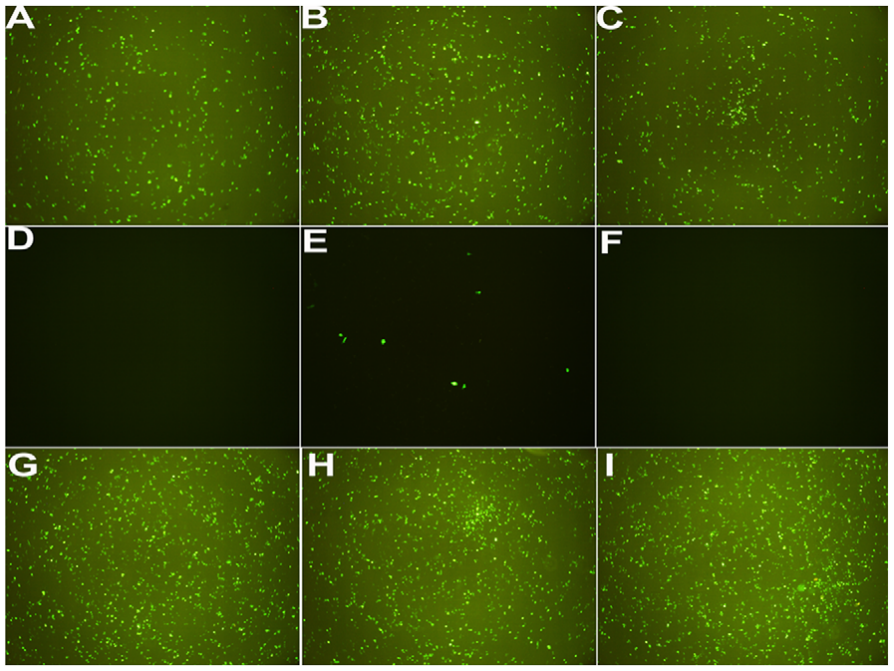
Representative fluorescent microscope images (40×) of A549 cells transfected by L1 at N/P ratio of 1.5 (A), 2 (B), 2.5 (C), L2 at N/P ratio of 2 (D), 2.5 (E), 3 (F) and L3 at N/P ratio of 8 (G), 10 (H), 12 (I). The lipid/DOPE ratios were 1 ∶ 1 and the concentration of DNA was 2 µg/well.

The in vitro transfection abilities of title lipids were initially studied by using green fluorescent protein reporter gene pEGFP-N1 as guest gene and A549 as target cells. In the experiments of **L1–L3** mediated GFP expression, N/P ratios were varied from 0.5 to 20 for all lipoplexes. Figure 6 shows the images taken under the N/P ratio range in which best results for each lipoplex were obtained. Results indicated that **L1** and **L3**-based lipoplexes could lead to obvious GFP expression at N/P ratio of 1.5–2.5 and 8–12 respectively, while **L2**-based lipoplexes was found to be nearly incompetent for transfection at all studied N/P ratios. For **L1** and **L3**, under the N/P ratios not shown in Figure 6, fewer cells that expressed GFP were observed by microscopy.

**Figure 7:**
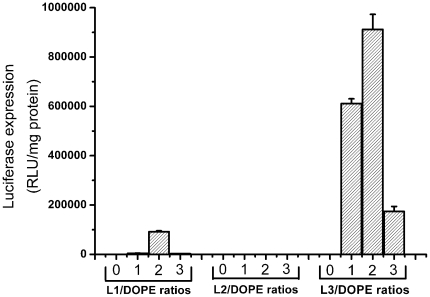
Transfection efficiencies of lipoplexes formed from L1–L3 at various lipid/DOPE ratios by keeping the N/P ratio at 2 (for L1 and L2) and 10 (for L3) in A549 cells. Concentration of the DNA was 2 µg/well. Data are expressed as relative light units (RLU)/mg of protein.

Besides chemical structure of cationic lipids and the N/P ratios, the mole ratio of cationic/neutral lipids in the liposome also has large influence on the transfection efficiency [33]. We carried out a luciferase transfection experiments by varying the mole ratios of the cationic lipids against DOPE at the N/P ratio of 2 (**L1** and **L2**) and 10 (**L3**) using pGL-3 plasmid DNA in A549 cells. As shown in Figure 7, lipoplexes formed from **L1** and **L3** were found to show maximum transfection efficiency at lipid/DOPE mole ratio of 1 ∶ 2, whereas **L2**-based lipoplexes still showed nearly no transfection across all lipid/DOPE ratios. The higher transfection efficiency could be due to cells expressing a higher level of the delivered gene or a greater proportion of cells being transfected, or a combination of both [34]. Since the numbers of cells transfected by **L1** and **L3** were found to be comparable (As shown in Figure 6), while **L3** showed appropriately 10 times higher transfection efficiency than that of **L1** under lipid/DOPE mole ratio of 1 ∶ 2, we speculated that **L3** might induce a higher level of the delivered gene into cells. In addition, **L1**-based lipoplexes was found to have no gene transfection activities at other lipid/DOPE ratio except 1 ∶ 2, and all lipoplexes exhibited no transfection in the absence of DOPE.

**Figure 8:**
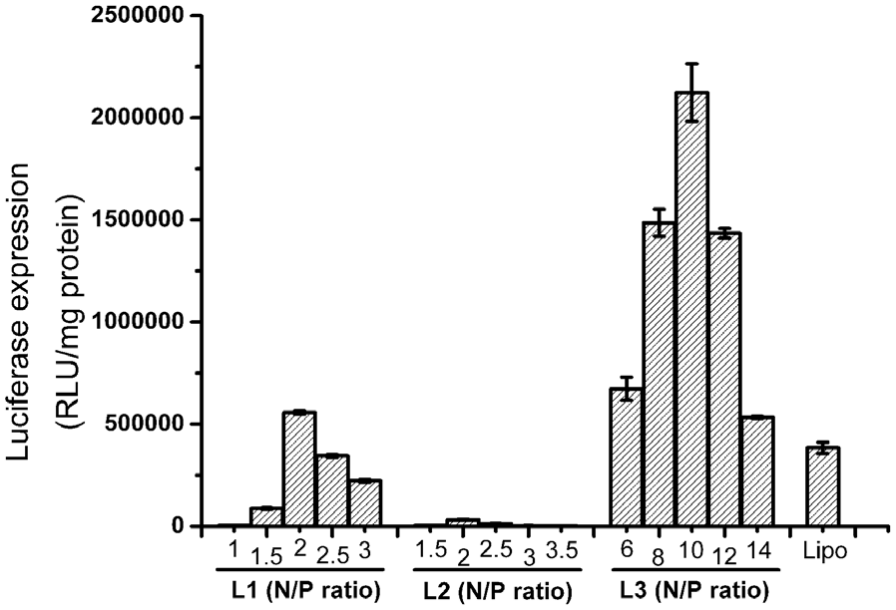
Transfection efficiencies of L1–L3 at various N/P ratios using optimized lipid/DOPE ratio of 1 ∶ 2 in A549 cells. Concentration of the DNA was 2 µg/well and lipofectamine 2000™ was used as control. Data are expressed as relative light units (RLU)/mg of protein.

After the confirmation of the optimum amount of DOPE (1 ∶ 2, **L1**–**L3**/DOPE), the effect of N/P ratios for these cationic lipids was further investigated by luciferase transfection experiments. Similar to the results of pEGFP-N1 assays, Figure 8 showed that **L1** and **L3**-based lipoplexes gave the best transfection efficiency at N/P ratio of 2 and 10, respectively. Meanwhile, **L2**-based lipoplexes only showed very low transfection activitiy at the N/P ratio of 2. Under the optimized conditions including DOPE amount and N/P ratio, the transfection efficiency of **L3** was 3.8 times higher than that of **L1** and was 5.5 times higher than that of the popular commercially available gene delivery agent Lipofectamine 2000™. Obviously, lipid **L3** was found to be the best effective gene delivery agent in this series of protonated cyclen and imidazolium salt-based single tail cationic lipids. For comparison between **L1** and **L3**, the main difference in their structures is that **L3** has an ester bond which not exists in the structure of **L1**. The two compounds also have similar DNA-binding ability, similar particle sizes and zeta potentials of their formed lipoplexes. The different gene transfection performance of **L1** and **L3** might be attributed to the change in the balance between hydrophilicity and hydrophobicity of the cationic lipids, which was originated from their different linker group and their different aggregation performance [32]. More interestingly, the sole structural difference between **L2** and **L3**, which led to their completely different gene transfection efficiencies, is the orientation of ester group. This result is consistent with that obtained by Rajesh et al [35] who demonstrated for the first time that even a minor structural variation such as linker orientation reversal in cationic lipids can profoundly influence DNA-binding characteristics, membrane rigidity, membrane fusibility, cellular uptake, and consequent gene delivery efficacies of the cationic liposomes and lipoplexes.

**Figure 9:**
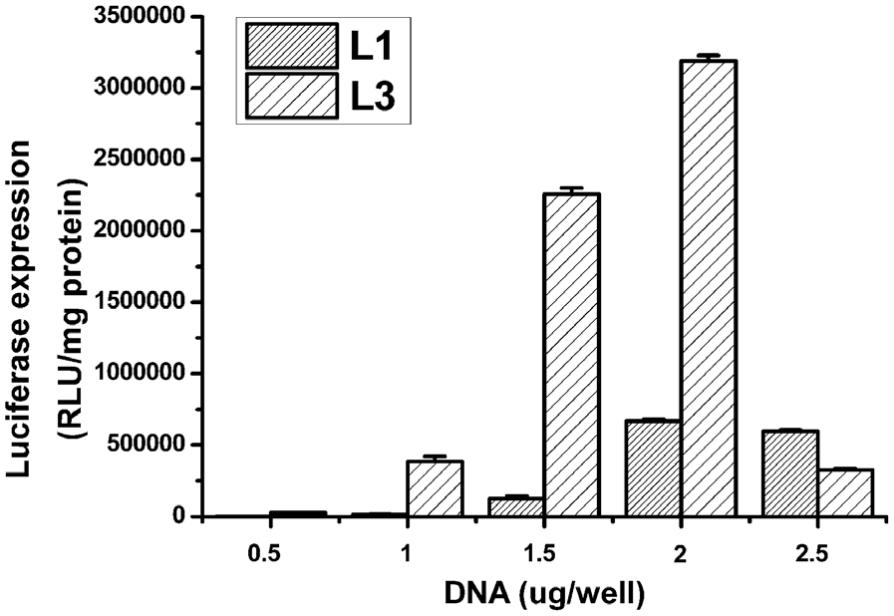
Transfection efficiencies of L1 and L3 at various amount of DNA using optimized lipid/DOPE ratio of 1 ∶2 (for L1 and L3) and optimized N/P ratio of 2 (for L1) and 10 (for L3) in A549 cells. Data are expressed as relative light units (RLU)/mg of protein.

It was reported that the amount of DNA used in transfection experiments may also affects the transfection efficiency of cationic lipids [32]. In all transfection experiment above, 2 µg/well of DNA was used. To see the effect of DNA amount on the transfection efficiency of these cationic lipids, we also performed luciferase transfection experiments using **L1** and **L3** at the optimized conditions. As shown in Figure 9, the DNA amounts were varied from 0.5 to 2.5 µg/well. Decreased transfection efficiencies were observed in the experiments using the amounts of DNA of 0.5, 1, 1.5 and 2.5 µg/well. When 0.5 µg/well of DNA was used, nearly no transfection activities for both of the lipids were found. These results indicated that 2 µg/well of DNA was the optimal DNA concentrations in the transfection employing **L1** and **L3**.

**Figure 10:**
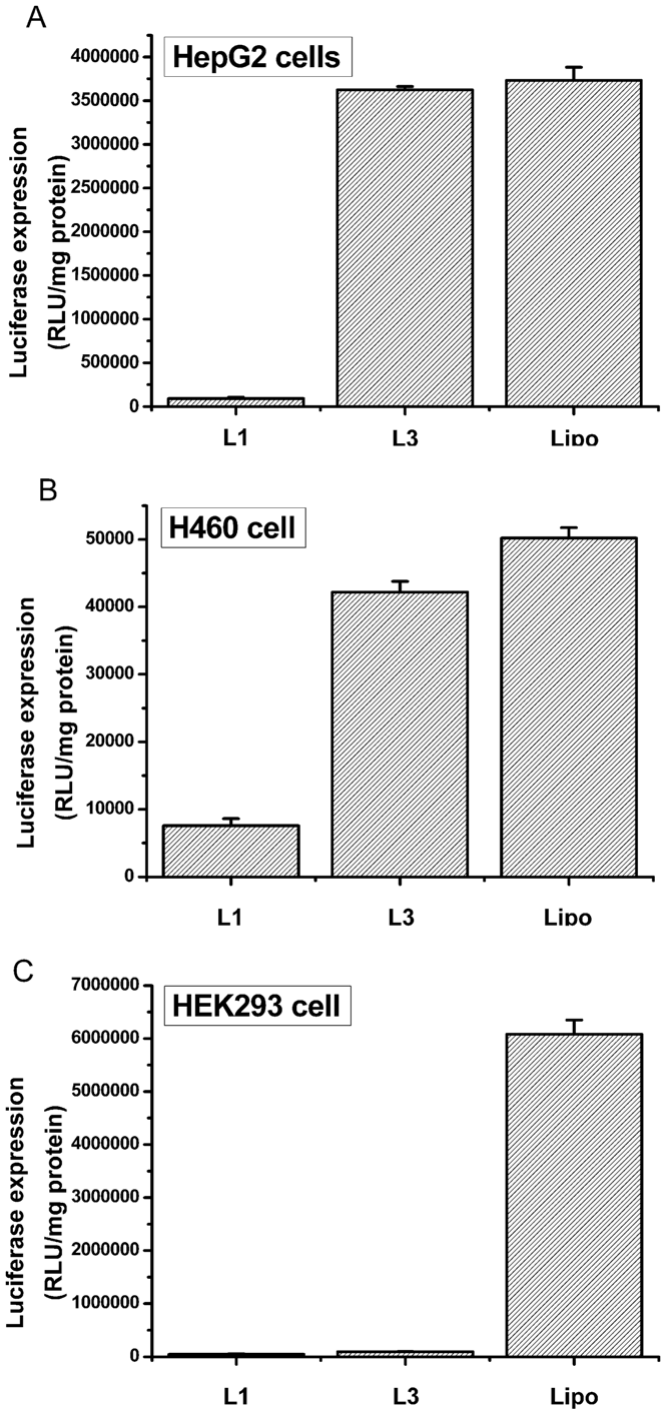
Transfection efficiencies of L1 and L3-based lipoplexes using optimized lipid/DOPE ratio of 1 ∶2 (for L1 and L3) and optimized N/P ratio of 2 (for L1) and 10 (for L3) in Hep G2 (A), H460 (B) and HEK293 (C) cells. Concentration of the DNA was 2 µg/well and lipofectamine 2000™ was used as control (Lipo). The data are expressed as relative light units (RLU)/mg of protein.

To further explore the transfection efficacy of **L1** and **L3**, luciferase reporter gene delivery studies were carried out in three other cell lines including Hep G2 (human hepatocellular carcinoma), H460 (human lung cancer cells) and HEK293 (human embryonic kidney cells). As shown in Figure 10A and 10B, **L3**-based lipoplexes showed comparable transfection efficiencies as to Lipofectamine 2000™ in cancer cells Hep G2 and H460, whereas **L1**-based lipoplexes only gave dramatically decreased transfection efficiencies, especially in Hep G2 cells. To our surprise, **L1** and **L3** were both found to be nearly futile in the transfection towards normal cells HEK293 (Figure 10C). These results gave us positive information that **L3** displayed tumor cell-targeting property, which still need further experiments to be confirmed. Anyway, **L3** was further proved to be a promising gene delivery reagent.

### Cytotoxicity

**Figure 11:**
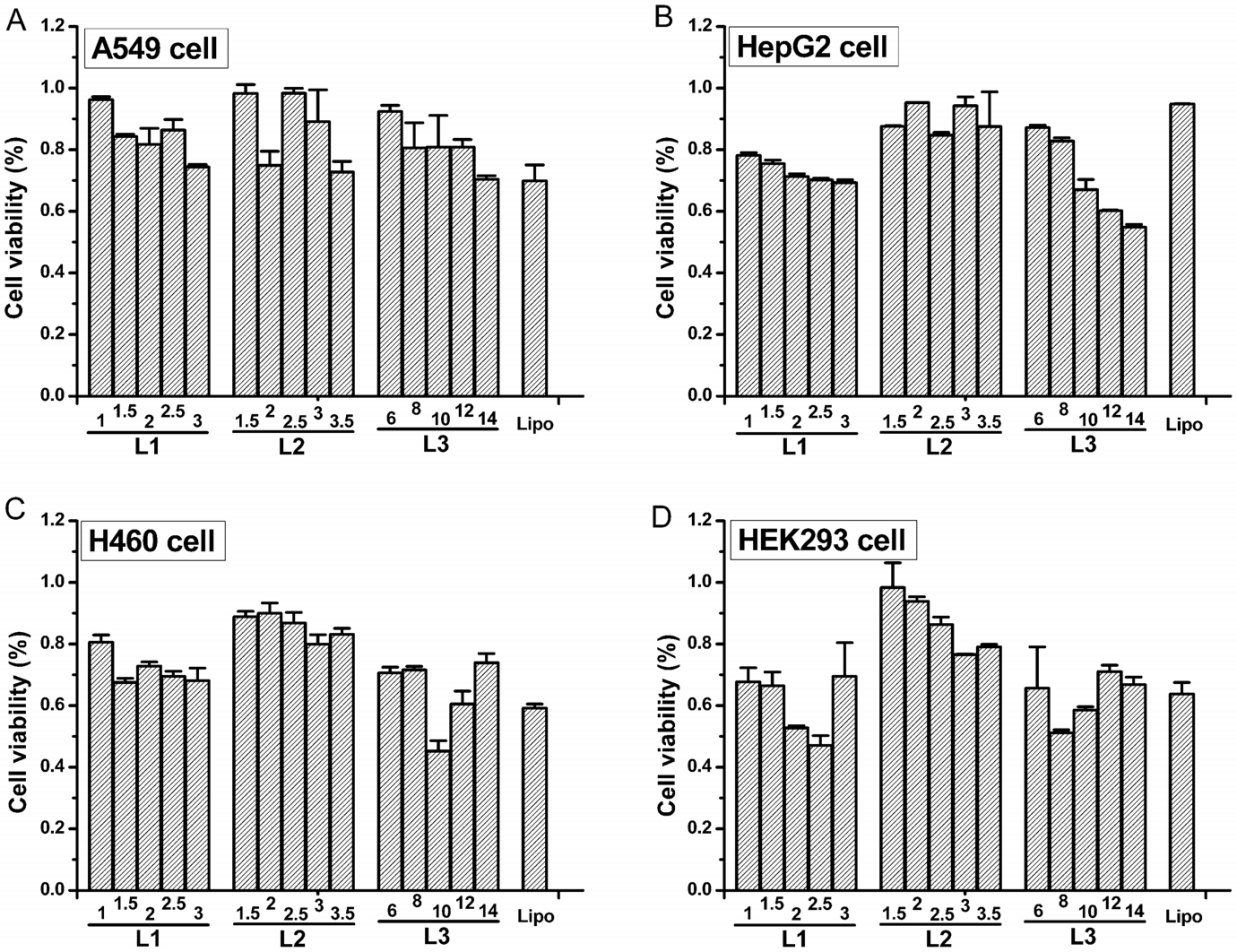
Cytotoxicity of L1-L3-based lipoplexes in A549 (A), Hep G2 (B), H460 (C) and HEK293 (D) cell lines using optimized lipid/DOPE ratio of 1 ∶ 2 at different N/P ratios (as indicated at the bottom of each bar). The concentration of DNA was 0.5 µg/well and the cytotoxicity of lipoplexes prepared from Lipofectamine 2000™ was used as control (Lipo).

MTT-based cytotoxicity studies were performed in all four studied cell lines using optimized DOPE amount at different N/P ratios. The cytotoxicity of lipoplexes formed from Lipofectamine 2000™, which was prepared based on the standard conditions specified by the manufacture, was used as control. As shown in Figure 11, for the four cell lines studied here, in most cases, the cell viability varied from 70% to 98%, which were similar to or slightly higher than that caused by Lipofectamine. The exception was found in Hep G2 cells, in which the cell viabilities of three lipid-based lipoplexes were slightly lower than Lipofectamine, especially at high N/P ratios. Among the three lipids, despite its poor transfection efficiency, the polyplex formed from **L2** displayed relatively lower cytotoxicity.

### Conclusion

In summary, three novel cationic lipids (**L1**–**L3**) with protonated macrocyclic polyamine (cyclen) and imidazolium salt as headgroups and single long chain as hydrophobic moieties were designed and easily prepared. In association with DOPE, **L1** and **L3** could well bind and condense DNA in to nanoparticles with the sizes of around 200 nm and zeta-potentials of 5–20 mV. TEM also showed well-shaped spherical particles formed from **L1**, **L3** and DNA. In contrast, **L2** could not bind to DNA well, which was confirmed by gel retardation assays, fluorescent quenching experiments, DLS and TEM. Of the three lipids, the lipoplex formed from **L3** was found with high efficiency in gene transfer in three tumor cell lines including A549, Hep G2 and H460. The optimized gene transfection efficacy of **L3** was nearly 5.5 times more efficient than that of the popular commercially available gene delivery agent Lipofectamine 2000™ in A549 cells. Meanwhile, **L1** and **L3** had nearly no gene transfection performance in normal cells HEK293, indicating that these lipids displayed tumor cell-targeting property, which still need further experiments to be confirmed. No significant cytotoxicity was found for the lipoplexes formed by **L1**–**L3** in four cell lines, and their cytotoxicities were similar to or slightly lower than the lipoplexes prepared from Lipofectamine 2000™. These studies may extend the application areas of macrocyclic polyamines, especially for cyclen. Further investigations including the deep-insight studies towards the cell-targeting delivery and more structure-activity relationship of this type of cationic lipids are now in progress.

## Supporting Information

Scheme S1 **Synthetic procedures of title compounds L1–L3.**
(TIF)
